# Evaluation of the functional role of corpus luteum cavity in recipient selection for bovine embryo transfer

**DOI:** 10.5713/ab.25.0518

**Published:** 2025-10-22

**Authors:** Jihyun Park, Ahmad Yar Qamar, Wonyou Lee, Kilyoung Song, Miyun Park, Leegon Hong, Seonggyu Bang, Younghye Ro, Sanghoon Lee, Minjung Kim, Junkoo Yi, Jongki Cho

**Affiliations:** 1 College of Veterinary Medicine and Research Institute for Veterinary Science, Seoul National University, Seoul, Korea; 2 College of Veterinary Medicine, Chungnam National University, Daejeon, Korea; 3 College of Veterinary and Animal Sciences, Jhang Sub-campus of University of Veterinary and Animal Sciences, Lahore, Pakistan; 4 SOBOM Co., Ltd., Hanam, Korea; 5 Farm Animal Clinical Training and Research Center, Institutes of Green-Bio Science and Technology, Seoul National University, Pyeongchang, Korea; 6 College of Veterinary Medicine, Kangwon National University, Chuncheon, Korea; 7 School of Animal Life Convergence Science, Hankyong National University, Anseong, Korea; 8 Gyeonggi Regional Research Center, Hankyong National University, Anseong, Korea

**Keywords:** Bovine, Corpus Luteum, Embryo Transfer, Pregnancy Rate, Progesterone, Recipient

## Abstract

**Objective:**

The effectiveness of bovine embryo transfer (ET) programs is significantly influenced by selection of optimal recipient cows, in which a functional corpus luteum (CL) is critical for the maintenance of pregnancy. The relationship between CL size and blood perfusion (CLBP) has been extensively studied; however, the implications of CL cavities (CL_cav_) on fertility remain controversial. This study aimed to assess the functional significance of CL_cav_ in the selection of recipients for ET by evaluating its association with pregnancy outcomes, CLBP, and hormonal profiles.

**Methods:**

Ninety-eight Hanwoo cows were subjected to estrus synchronization and evaluated using transrectal ultrasonography. Eighty-five recipients were selected based on CL diameter (≥15 mm) and the absence of large dominant follicles with 10 mm or more. On Day 6.5 post-estrus, CL type (compact vs. cavitary), CLBP (color Doppler), and hormone levels (estradiol [E_2_] and progesterone [P_4_]) were recorded. ET was performed using *in vitro*-produced fresh or vitrified embryos, with pregnancy status assessed 40–60 days following transfer.

**Results:**

CL_cav_ identified in 18.8% of recipients, but pregnancy rates were not significantly different between cows with and without CL_cav_ (50.0% vs. 62.3%, p>0.05). When CL_cav_ occupied ≥40% of the CL volume (with CL_cav_ diameter 18 mm or less), conception rates improved (62.5% vs. 37.5%), in conjungtion with elevated E_2_ levels. At CLBP levels of 40% or higher, both CL_cav_ size and P_4_ concentration reached their peak, but excessive CLBP did not enhance conception rates.

**Conclusion:**

These findings suggest that CL_cav_ does not negatively affect luteal function or pregnancy outcomes and may, in some cases, indicate enhanced luteal activity. Integrating CL morphology, vascularization, and hormonal balance into recipient selection criteria could improve ET efficiency. Future studies should explore the physiological mechanisms that contribure to CL_cav_ formation and its role in reproductive success.

## INTRODUCTION

Embryo transfer (ET) is a widely used reproductive biotechnology in the bovine industry, playing a pivotal role in genetic improvement and herd management by increasing reproductive efficiency and the dissemination of elite genetics [1,2]. While advancements in ET techniques have improved conception rates, several factors continue to influence its success. Among these, the physiological and endocrine status of the recipient cow at the time of ET is a critical determinant of pregnancy establishment and maintenance [3–5]. The corpus luteum (CL), a transient endocrine structure formed after ovulation, is a key component of this process due to its role in progesterone (P_4_) secretion, which is essential for endometrial receptivity and embryo survival [6–8]. Consequently, selection criteria for recipient cows have historically relied on CL presence, size, and functional status as primary indicators of uterine readiness for implantation [3,9].

Recent advances in ultrasonographic imaging have facilitated a more detailed evaluation of the CL, revealing morphological variations such as CL cavities (CL_cav_), also referred to as central lacunae, which are fluid-filled spaces within the CL [10]. While CL size and blood perfusion have been correlated with P_4_ production and pregnancy outcomes, the impact of CL_cav_ on fertility remains a topic of debate. Some studies suggest that the presence of CL_cav_ may be indicative of incomplete luteinization or suboptimal luteal function, potentially leading to reduced P_4_ production and lower pregnancy rates [11,12]. However, conflicting evidence exists, with reports indicating that CL_cav_ may not negatively affect conception rates and, in some cases, may even be associated with enhanced luteal activity [6]. A recent study assessing recipient cows in a large-scale ET program found no significant differences in conception rates between cows with CL_cav_ and those with CL_com_, suggesting that, regardless of the type of CL that is compact or has a cavity, other factors may be predictive factors that are related to reproductive success [12–14].

In addition to CL morphology, other ovarian parameters, including the presence of dominant follicles (DF), serum estradiol (E_2_) and P_4_ concentrations, and CL blood perfusion (CLBP), have been investigated as potential predictors of successful conception [15,16]. The balance between E_2_ and P_4_ plays a crucial role in modulating uterine receptivity, and an elevated E_2_/P_4_ ratio has been associated with poor pregnancy outcomes in ET programs [17,18]. Recent studies indicate that optimal hormonal balance may be more predictive of pregnancy success than absolute E_2_ or P_4_ concentrations alone [18,19]. Furthermore, while increased CLBP is generally considered a sign of enhanced luteal function, excessive vascularization may not always translate to improved fertility [20]. Although increased CLBP is generally associated with improved reproductive outcomes, some reports suggest that excessive vascularization could reflect abnormal luteal activity, warranting further investigation into standardized CLBP assessment criteria [21,22]. Understanding the interactions between these factors is essential for optimizing recipient selection criteria and improving ET efficiency.

Despite the wealth of research on CL function and its relationship to fertility, there is limited information regarding the specific role of CL_cav_ in ET outcomes, particularly in Hanwoo cattle (native to Korea). Given the economic and genetic significance of ET programs, refining recipient selection protocols based on a comprehensive assessment of ovarian morphology and hormonal status is imperative. This study aims to evaluate the impact of CL_cav_ presence and size on pregnancy rates in ET recipient cows and to investigate the relationship between CL_cav_, CLBP, and serum hormone levels. By integrating multiple ovarian parameters, we seek to determine whether CL_cav_ should be considered a critical factor in recipient selection and to provide insights that could enhance the success of bovine ET programs.

## MATERIALS AND METHODS

### Animals and estrus synchronization

**Figure 1:**
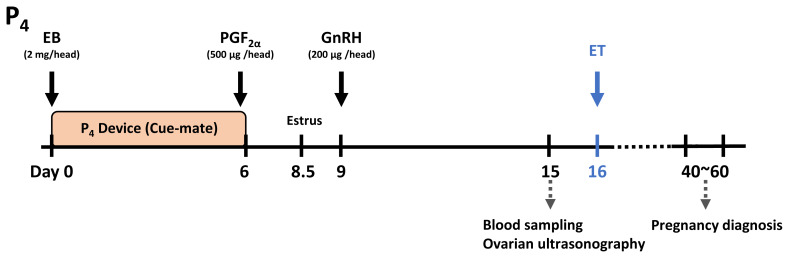
Schematic representation of the J-Synch protocol for estrus synchronization in embryo transfer (ET) recipient cows, including ultrasonographic monitoring of ovarian status and measurement of serum progesterone (P_4_) and estradiol (E_2_) concentrations. EB, estradiol benzoate; PGF_2α_, prostaglandin F_2α_; GnRH, gonadotropin-releasing hormone; P_4_ device (Cue-mate), an intravaginal device containing 1.56 g of progesterone.

This study was performed as part of a commercial ET program in Hongcheon-gun, Gangwon-do, Korea, with the participation of 11 farms. A total of 98 Hanwoo recipient cows were included in the study, comprising 21 nulliparous and 77 multiparous cows. The experiment was performed between April and September 2024. Estrus synchronization followed the J-Synch protocol (Figure 1), which involved inserting an intravaginal P_4_ device (Cue-Mate; Bioniche Animal Health Australia/Asia and Bayer Animal Health) containing 1.56 g of P_4_ on Day 0. Simultaneously, 2.0 mg of intramuscular estradiol benzoate (Esrone; Samyang Anipharm) was administered. On Day 6, the intravaginal device was removed, and 500 μg of prostaglandin F_2α_ (PGF_2α_; Synchromate; Pfizer) was injected intramuscularly. Estrus was detected approximately 2.5 days after the PGF_2α_ injection, and 200 μg of gonadotropin-releasing hormone (GnRH; Gonadon; Dong Bang) was administered intramuscularly 12 h after estrus detection.

### Ovarian assessment and ultrasonography

**Figure 2:**
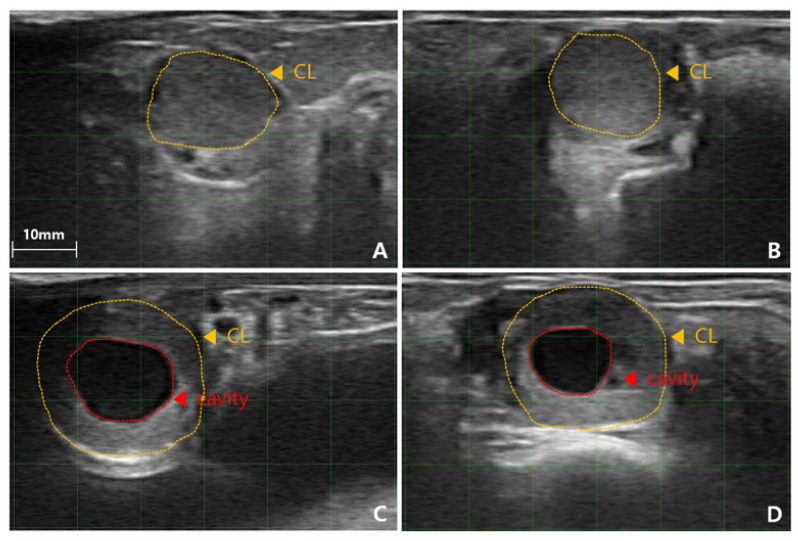
Ultrasound image showing the corpus luteum (CL) of a bovine ovary one day before ET. (A–D) Images in mode B (brightness; gray scale). (A, B) Compact CL. (C, D) Cavity CL. ET, embryo transfer.

**Figure 3:**
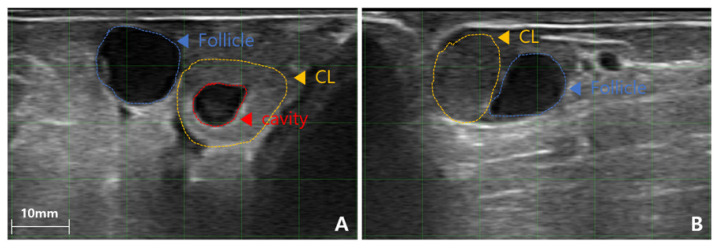
Ultrasound image showing dominant follicle coexisting with corpus luteum (CL) in a bovine ovary one day before ET. (A, B) Images in mode B (brightness; gray scale). (A) Dominant follicle coexisting with cavity CL. (B) Dominant follicle coexisting with compact CL. ET, embryo transfer.

**Figure 4:**
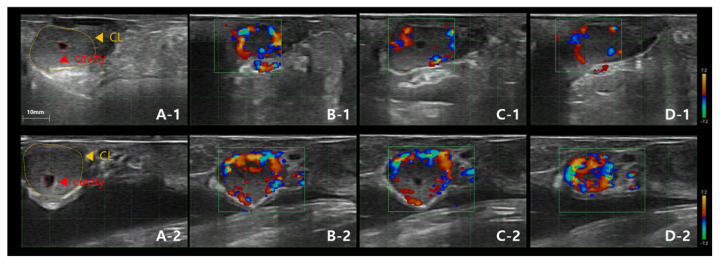
Ultrasound image showing corpus luteum blood perfusion (CLBP) in a bovine ovary one day before ET. (A-1, A-2) Images in mode (brightness; gray scale). (B-1–D-1, B-2–D-2) Images in Color-Doppler mode (detection limit: 7.2 m/s). (A-1, A-2) Corpus luteum (CL). (B-1, B-2) CLBP on the left side of the CL. (C-1, C-2) CLBP in the center of the CL. (D-1, D-2) CLBP on the right side of the CL. In image A (A1, A2), the CL cavity is small; images B (B1, B2) and D (D1, D2) show the left and right sides, where the CL cavity is not visible. The CL cavity is visible only in C, which is the exact center of the CL. ET, embryo transfer.

Transrectal ultrasonography was performed on all synchronized cows 6.5 days post-estrus using a 6.5 MHz linear probe (SR-1C; Sonoptek). One experienced technician performed the ultrasonographic evaluation to ensure consistency in data collection. The CL was categorized as either a (CL_com_ or a CL_cav_, based on B-mode ultrasound imaging. The diameter of the CL, any CL cavity present, and the DF size were recorded (Figures 2, 3). CL diameter was measured as the mean of its length and width at the center, with CL_com_≥15 mm and CL_cav_≥3 mm considered eligible for ET. DF presence and size (≥10 mm) were also documented. CLBP was assessed using color Doppler ultrasonography (SR-1C; Sonoptek), which allowed visualization of vascularization within the CL (Figure 4). The CLBP was visually estimated as a percentage of the luteal area displaying blood flow. Cows with a CL diameter<15 mm or an absent CL were excluded from ET (n = 13). Due to technical limitations, excessive cow movement, or temporary ultrasonography errors, CLBP measurements were not recorded for 11 cows.

### Blood sampling and hormonal analysis

Blood samples were collected from the jugular vein using lithium heparin tubes (BD Vacutainer) on Day 6.5 post-estrus. The plasma was separated within 2 h of collection via centrifugation at 2,000 ×g for 15 min and stored at −70°C until analysis. Prior to hormonal analysis, frozen plasma samples were thawed at room temperature. P_4_ and E_2_ concentrations were quantified using commercial enzyme-linked immunosorbent assay (ELISA) kits (NBP2-60122, Novus Biologicals; 501890, Cayman Chemical). To improve the accuracy of E_2_ measurements, samples were pre-extracted with methanol (322415; Sigma-Aldrich) and concentrated using a nitrogen concentrator (NDK200-1N; LabTech).

### Embryo transfer procedure

Embryos used in this study were produced by *in vitro* fertilization (IVF) using oocytes obtained via ovum pick-up (OPU) from donor Hanwoo cows. Oocytes were matured *in vitro* (IVM) in TCM-199 medium (Gibco) supplemented with 5% bovine serum albumin (BSA) for 22 h. Fertilization was performed with semen from a Korean Proven Bull (KPN) using the IVF medium (VitroFert; ART Lab Solutions). The embryos were cultured for total of seven days in cleavage medium (VitroCleave; ART Lab Solutions) for five days and blastocyst medium (VitroBlast; ART Lab Solutions) for two days before either being transferred fresh or cryopreserved through vitrification. For vitrified embryos, the thawing protocol was applied before transfer [23]. Recipient cows underwent ultrasonographic evaluation one day prior to ET. Those with a CL diameter<15 mm or an excessively large follicle relative to the CL were excluded from ET (n = 13). A total of 52 fresh embryos and 33 vitrified-thawed embryos were transferred. ET was performed by two skilled technicians using a 0.25 mL ET gun (Watanabe Tecnologia Aplicada) and a sterile ET sheath (Minitube). Each embryo was deposited into the uterine horn ipsilateral to the ovary bearing the CL.

### Pregnancy diagnosis

**Figure 5:**
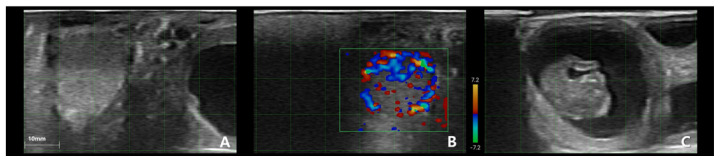
The ultrasound image shows the corpus luteum and fetus on day 56. (A, C) Images in mode (brightness; gray scale). (B) Image in Color-Doppler mode (detection limit: 7.2 m/s). (A) Corpus luteum; (B) CLPB (corpus luteum blood perfusion) in the center of the corpus luteum; (C) fetus.

Pregnancy status was determined via transrectal ultrasonography 40–60 days post-ET using a 6.5 MHz linear probe (SR-1C; Sonoptek). Successful pregnancies were defined based on the presence of an intact CL, visible amniotic fluid, and a developing fetus (Figure 5). The conception rate was calculated as the number of confirmed pregnancies divided by the total number of ETs performed.

### Statistical analysis

All statistical analyses were performed to evaluate differences in ovarian parameters and hormonal profiles among the various experimental groups. For comparisons involving multiple groups, one-way analysis of variance (ANOVA) was performed to identify overall differences, followed by Tukey’s post hoc test to determine pairwise significance. For comparisons between two groups, an independent t-test was used. Given the variability in group sizes, an equal variance test was conducted prior to the t-test to ensure homogeneity of variances. The comparison of conception rates was performed using the Chi-square test (GraphPad Prism 8.0.1; non-pregnant, 0; pregnant, 1). All statistical analyses except conception rate were performed using SPSS 29 (IBM SPSS Statistics 29; IBM), and the results were expressed as the mean±standard error of the mean (SEM). Statistical significance was determined at a p-value threshold of <0.05.

## RESULTS

### Presence of corpus luteum cavities and pregnancy outcomes

Among the 85 recipient cows selected for ET, 16 (18.8%) exhibited a detectable CL_cav_, whereas the remaining 69 (81.2%) had CL_com_. The CL_cav_ group had a slightly larger CL diameter (21.5±0.8 mm) compared to the CL_com_ group (19.3±2.8 mm), although this difference was not statistically significant. Similarly, serum P_4_ concentrations were slightly higher in the CL_cav_ group (37.7±3.5 ng/mL) than in the CL_com_ group (33.0±2.2 ng/mL; p>0.05). Likewise, there were no differences in ovarian morphology and hormone levels, and there was no significant difference in pregnancy rate between cows with and without CL_cav_ (50.0% vs. 62.3%, p>0.05) (Table 1). These results indicate that the presence of CL_cav_ does not have a detrimental effect on pregnancy outcomes and suggests that its impact on luteal function may be more complex than previously assumed.

### Size of corpus luteum cavities and its association with pregnancy success

To further evaluate the functional significance of CL_cav_, recipient cows were categorized based on the proportion of CL_cav_ relative to the total CL volume: those with CL_cav_ occupying 40% or more of the CL and those with CL_cav_ occupying less than 40%. Cows in which CL_cav_ comprised 40% or more of the total CL volume exhibited a higher pregnancy rate (62.5% vs. 37.5%). Although this difference was not statistically significant (p = 0.62), the odds of conception tended to be higher in cows with larger CL_cav_ (OR = 2.78, 95% CI: 0.37–21.03). In addition, these cows had higher serum E_2_ concentrations (83.8±21.8 pg/mL) compared to cows with smaller CL_cav_ (48.0±6.5 pg/mL) but no significant difference was observed in the data (p>0.05) (Table 2). These findings suggest that a larger CL_cav_ may not necessarily indicate impaired luteal function but could instead reflect a compensatory physiological response that supports reproductive success. The increased E_2_ levels in cows with larger CL_cav_ may contribute to improved endometrial receptivity, thereby enhancing pregnancy outcomes.

### Relationship between corpus luteum blood perfusion and corpus luteum cavities

CLBP was analyzed to determine whether vascularization differed between CL_cav_ and CL_com_. Cows with CL_cav_ exhibited higher P_4_ concentrations when CLBP exceeded 40%, suggesting an association between increased luteal vascularization and enhanced P_4_ secretion. However, the highest pregnancy rates were observed in cows with moderate CLBP (<25%) rather than in those with excessive perfusion (71.4% vs. 50.0%) (Table 3). This finding indicates that while an increase in CLBP may support luteal function by enhancing P_4_ synthesis, excessive vascularization does not necessarily lead to improved pregnancy rates. An optimal balance of luteal blood flow may be required to sustain a favorable uterine environment for embryo implantation.

### Interaction between corpus luteum cavities, hormonal profiles, and pregnancy success

Given the importance of hormonal balance in pregnancy establishment, further analysis was conducted to evaluate the relationship between CL_cav_, E_2_/P_4_ ratio, and pregnancy outcomes. The E_2_/P_4_ ratio tended to be higher in cows with CL_com_ (3.4±2.0) compared to those with CL_cav_ (2.0±0.4), although this difference was not statistically significant (p>0.05) (Table 1). However, when a CL_cav_ was present, the E_2_/P_4_ ratio was higher and conception rates were also increased when CL_cav_ occupied more than 40% of the CL volume (Table 2). These findings suggest that while the presence of CL_cav_ alone does not negatively impact pregnancy outcomes, its functional role may be dependent on its interaction with other ovarian parameters, particularly hormonal dynamics.

## DISCUSSION

The selection of suitable recipient cows is a critical factor influencing the success of ET programs, as the physiological status of the recipient at the time of ET directly affects conception rates and overall reproductive outcomes. Traditionally, the presence of a functional CL has been a key criterion for recipient selection, given its role in producing P_4_, which is essential for pregnancy maintenance [6]. However, additional factors such as the presence and size of a CL_cav_, CLBP, and hormonal balance have been increasingly recognized as important determinants of reproductive success. Recent studies have also suggested that the intensity of estrus expression in recipient cows may be associated with higher pregnancy rates, indicating that behavioral and endocrine factors should be considered alongside ovarian morphology [24]. This study aimed to assess the combined impact of these factors on pregnancy outcomes in Hanwoo cows undergoing ET, thereby improving selection criteria in commercial settings.

The presence of CL_cav_ in recipient cows has been a subject of debate in reproductive research, with conflicting reports on its influence on conception rates. Some studies have suggested that CL_cav_ indicates compromised luteal function and reduced P_4_ production, potentially leading to lower pregnancy rates [6,25]. However, our findings align with previous research indicating that the presence of CL_cav_ does not necessarily impair reproductive success [3]. In our study, conception rates did not significantly differ between cows with and without CL_cav_, suggesting that a CL with a central cavity can still function adequately to support early pregnancy. Interestingly, when CLcav occupied more than 40% of the total CL volume, conception rates were higher compared to those with smaller CLcav. However, given the limited sample size in this study, caution is needed when interpreting these findings. Although this difference did not reach statistical significance (p = 0.62), the odds of conception tended to be higher in cows with larger CLcav (OR = 2.78, 95% CI: 0.37–21.03). This trend suggests a potential positive association that warrants further confirmation in studies with larger sample sizes. As conception rate is a qualitative variable, a larger sample size would be necessary to achieve statistical significance and validate these results. Future studies with an expanded cohort are warranted to confirm the impact of CLcav size on reproductive success in bovine ET recipients [6,26]. This observation suggests that, rather than being a negative indicator, the presence of a larger CL_cav_ may reflect an active luteal response that compensates for the cavity. Previous studies have indicated that luteal cells surrounding the cavity may become hyperfunctional, producing sufficient P_4_ to maintain pregnancy [27]. Moreover, recent research has shown that metabolic parameters influence CL_cav_ formation and luteal function, suggesting that a more comprehensive assessment of recipient metabolic status may refine selection criteria [12]. This compensatory mechanism may explain the increased conception rates observed in cows with larger CL_cav_ in this study.

CLBP is considered a reliable indicator of luteal function, as it reflects the vascularization and functional capacity of the CL [16,20,28]. In this study, we observed a notable interaction between CL_cav_ and CLBP, with cows exhibiting larger CL_cav_ also demonstrating higher CLBP. This finding suggests that CL_cav_ may be associated with increased blood perfusion, potentially enhancing luteal activity and P_4_ synthesis [29]. These results are in agreement with previous studies reporting that well-perfused CLs with high blood flow tend to exhibit superior reproductive performance due to improved nutrient and hormone delivery [6,30]. However, it is also essential to consider that excessive vascularization may indicate abnormal luteal activity, as observed in other species, where excessive blood flow can contribute to local inflammation and disrupt pregnancy maintenance [29].

However, despite the positive correlation between CLBP and P_4_ levels, conception rates did not increase proportionally with CLBP. The highest conception rates were observed in cows with moderate CLBP coverage (<25%), while excessively high CLBP (>40%) did not provide additional benefits [25]. This suggests that while increased vascularization is beneficial, excessive blood flow may indicate abnormal luteal function or inflammation, potentially disrupting pregnancy establishment. Future studies should explore optimal CLBP thresholds to refine recipient selection criteria. Additionally, studies on hormonal modulation of CL function have shown that P_4_ supplementation during the early luteal phase can enhance CL development and function, potentially benefiting cows with suboptimal CLBP [29]. This approach could be explored further to optimize pregnancy rates in ET programs.

The findings of this study highlight the complexity of recipient selection in bovine ET programs. While conventional selection methods have focused primarily on CL size and P_4_ levels, our results suggest that additional factors such as CL_cav_ characteristics and CLBP should be considered when evaluating recipient suitability. Importantly, our data indicate that the presence of CL_cav_ should not be an exclusion criterion for ET, as CLs with larger cavities can still support pregnancy effectively [31]. This is further supported by recent findings in Holstein Friesian cows, where no significant differences in pregnancy outcomes were found between cows with CL_com_ and CLcav, emphasizing the importance of overall luteal function rather than morphology alone [32]. Furthermore, while CLBP serves as a useful marker of luteal activity, extreme variations in blood perfusion may not always translate to improved conception rates [33]. These insights have practical implications for commercial ET programs, where optimizing recipient selection is essential for maximizing pregnancy success rates. By adopting a more holistic approach that incorporates CL morphology, vascularization, and hormonal balance, practitioners can enhance the precision of recipient selection, ultimately improving reproductive efficiency in cattle operations.

While this study provides valuable insights into the role of CL characteristics in ET outcomes, certain limitations should be acknowledged. The study was conducted under commercial field conditions, introducing variability in factors such as recipient parity, nutritional status, and farm management practices [34]. Additionally, while CLBP assessment was performed using color Doppler ultrasonography, variations in operator interpretation may have influenced the results [6,35,36]. Future research should aim to standardize CLBP assessment protocols and explore the use of advanced imaging techniques, such as three-dimensional Doppler ultrasonography, for more accurate evaluations [29]. Moreover, while this study focused primarily on ovarian parameters, other factors such as endometrial receptivity, immune response, and embryo quality may also play crucial roles in pregnancy establishment. Recent studies have indicated that delayed endometrial preparation in heifers with high antral follicle counts may improve pregnancy rates, suggesting that incorporating endometrial and ovarian reserve assessments could further optimize recipient selection [37]. Integrating these factors into a comprehensive selection model could further improve ET success rates.

## CONCLUSION

In conclusion, this study demonstrates that CL_cav_ and CLBP are important factors influencing reproductive success in ET recipient cows. The presence of CL_cav_ does not negatively impact conception rates, and in some cases, larger CL_cav_ may be associated with improved pregnancy outcomes. Additionally, while increased CLBP reflects enhanced luteal function, an optimal range of perfusion is necessary for maximizing conception rates. These findings underscore the need for a multifaceted approach to recipient selection, incorporating CL morphology, vascularization, and hormonal balance to optimize ET outcomes. Future studies should further investigate these relationships using standardized methodologies to refine selection criteria and improve reproductive efficiency in commercial bovine ET programs.

## Figures and Tables

**Table 1:** Hormone levels, corpus luteum (CL) diameter and conception rate according to presence of CL with cavity

Groups	P_4_ concentration (ng/mL)1)	E_2_ conentraion (pg/mL)1)	E_2_/P_4_ ratio1)	CL (mm)1)	Conception rate (%)
CL compact	33.0±2.2	83.3±8.7	3.4±2.0	19.3±2.8	43/69 (62.3)
CL cavity	37.7±3.5	65.9±12.5	2.0±0.4	21.5±0.8	8/16 (50.0)

1) Data presented as mean±standard error of the means.

P_4_, progesterone; E_2_, estradiol.

**Table 2:** Comparison of corpus luteum (CL) diameter, hormone profiles, and conception rates according to the ratio of CL cavity

CL cavity (%)	CL (mm)1)	P_4_ concentration (ng/mL)1)	E_2_ concentration (pg/mL)1)	E_2_/P_4_ ratio1)	Conception rate (%)2)
<40	21.0±1.2	38.1±4.6	48.0±6.5	1.5±0.3	3/8 (37.5)
≥40	22.0±1.1	37.4±4.9	83.8±21.8	2.5±0.8	5/8 (62.5)

1) Data presented as mean±standard error of the means.

2) Although the difference in conception rate was not statistically significant (p = 0.62), cows with CL_cav_ occupying ≥40% of the CL volume tended to have higher conception rates (62.5% vs. 37.5%; OR = 2.78, 95% CI: 0.37–21.03).

P_4_, progesterone; E_2_, estradiol.

**Table 3:** Corpus luteum (CL) cavity diameter, hormone profiles and conception rate according to presence of corpus luteum blood perfusion (CLBP)

CLBP (%)	CL cavity (mm)1)	E_2_ concentration (pg/mL)1)	P_4_ concentration (ng/mL)1)	E_2_/P_4_ ratio1)	Conception rate (%)
<25	9.3±1.1	82.9±13.5	36.4±4.4	3.6±0.9	15/21 (71.4)
25–39	6.2±1.5	86.3±10.4	32.2±3.4	3.7±0.8	15/29 (51.7)
≥40	10.8±4.3	75.7±13.8	39.9±4.3	2.2±0.4	10/16 (62.5)

1) Data presented as mean±standard error of the means.

E_2_, estradiol; P_4_, progesterone.

## Data Availability

Upon reasonable request, the datasets of this study can be available from the corresponding author.
